# The Association of *ERBB2*-Low Expression With the Efficacy of Cyclin-Dependent Kinase 4/6 Inhibitor in Hormone Receptor–Positive, *ERBB2*-Negative Metastatic Breast Cancer

**DOI:** 10.1001/jamanetworkopen.2021.33132

**Published:** 2021-11-05

**Authors:** Kelvin K. H. Bao, Leone Sutanto, Shirley S. W. Tse, Ka Man Cheung, Jeffrey C. H. Chan

**Affiliations:** 1 Department of Clinical Oncology, Queen Elizabeth Hospital, Hong Kong

## Abstract

This cohort study investigates the association between low levels of *ERBB2* expression and progression-free survival among patients with HR+/*ERBB2*− metastatic breast cancer treated with CDK4/6 inhibitors.

## Introduction

Among the major breakthroughs in the treatment of hormone receptor–positive, human epidermal growth factor receptor 2–negative (HR+/*ERBB2*− [formerly *HER2*−]) metastatic breast cancer (MBC), the introduction of 3 cyclin-dependent kinase 4 and 6 (CDK4/6) inhibitors into the treatment arsenal has been associated with significant gains in progression-free survival (PFS) and overall survival of patients in first-line and second-line settings.^1^ However, phenotypic and genetic analysis has yet to identify robust predictive markers associated with the efficacy of these treatments. A potential marker candidate could be associated with the newly proposed subgroup with the nomenclature of *ERBB2*-low (formerly *HER2*-low), defined as tumors with an *ERBB2* immunohistochemistry (IHC) score of 1+ or 2+ with negative in situ hybridization assay. Approximately 50% of all breast cancers fall under such definition. Studies have suggested bidirectional crosstalk between *ERBB2* and HR pathways as a potential mechanism of hormonal resistance and poor outcomes.^2^ However, an association between *ERBB2*-low status and treatment outcomes of CDK4/6 inhibitors among patients with MBC was not established. We investigated the association between low levels of *ERBB2* expression and clinical outcomes among patients with HR+/*ERBB2*− MBC treated with CDK4/6 inhibitors.

## Methods

This cohort study was approved by the Kowloon Central/Kowloon East Research Ethics Committee, Hospital Authority, Hong Kong, which approved a waiver of informed consent because the data were deidentified. We followed the Strengthening the Reporting of Observational Studies in Epidemiology (STROBE) reporting guideline.

We identified consecutive patients with HR+/*ERBB2*− MBC who received CDK4/6 inhibitors with letrozole or fulvestrant from March 2017 to June 2020 from a single institutional cancer registry in Hong Kong. *ERBB2*-low expression was defined as an IHC score of 1+ or 2+ with a negative in situ hybridization. PFS was defined as the time from the initiation of CDK4/6 inhibitor to the date of radiological or clinical progression or death. The association between *ERBB2* expression levels and PFS was evaluated using log-rank test and multivariable Cox regression modeling. Covariates included the line of treatment, progesterone receptor status, and disease extent. An α level of 2-sided *P* ≤ .05 denoted statistical significance. Statistical analyses were performed using SPSS statistical software version 23.0 (IBM). Data were analyzed from July 2020 through January 2021.

## Results

There were 106 women with MBC eligible for analysis. The median (range) age at treatment was 58.0 (23.0-91.4) years. Most patients received palbociclib (90 patients [84.9%]), while the rest received ribociclib (16 patients [15.1%]). CDK4/6 inhibitor was used as the first-line treatment in 54 patients (50.9%). Most patients had tumors that were of ductal histology (88 patients [83.0%]), had a high estrogen receptor H score (ie, H ≥ 200; 76 patients [71.7%]), and had progesterone receptor positive status (81 patients [76.4%]). There were 24 patients [22.6%] with bone-only disease (Table).

**Table.** Demographic and Clinical Characteristics

Characteristic	Patients, No. (%)		
	Total (N = 106)	*ERBB2*-low (n = 82)^a^	*ERBB2* IHC score 0 (n = 24)^a^
Age, y			
Median (range)	58.0 (23.0-91.4)	58.6 (23.0-91.4)	57.0 (35.7-73.6)
<65	81 (76.4)	62 (75.6)	19 (79.2)
≥65	25 (23.6)	20 (24.4)	5 (20.8)
Disease status			
De novo	47 (44.3)	40 (48.8)	10 (41.7)
Relapse	59 (55.7)	42 (51.2)	14 (58.3)
Disease site			
Bone only	24 (22.6)	19 (23.1)	5 (20.8)
Visceral	59 (55.7)	46 (56.1)	12 (50.0)
Line of treatment			
First line	54 (50.9)	39 (47.6)	15 (62.5)
Second or third line	27 (25.5)	21 (25.6)	6 (25.0)
Estrogen receptor (H score)^b^			
≥200	76 (71.7)	60 (73.2)	15 (62.5)
<200	27 (25.5)	18 (22.0)	9 (37.5)
Progesterone receptor status			
Positive	81 (76.4)	63 (76.8)	18 (75.0)
Negative	25 (23.6)	19 (23.2)	6 (25.0)
CDK4/6 inhibitor			
Ribociclib	16 (15.1)	14 (17.1)	2 (8.3)
Palbociclib	90 (84.9)	68 (82.9)	22 (91.7)

Abbreviations: CDK, cyclin dependent kinase; IHC, immunohistochemistry.

^a^ Formerly *HER2*.

^b^ Data on estrogen receptor H score were unavailable for 3 patients.

There were 82 patients (77.3%) considered *ERBB2*-low expressing, which was associated with a shorter median PFS compared with 24 patients with *ERBB2* IHC score of 0 (8.9 months; 95% CI, 6.49-11.30 months vs 18.8 months; 95% CI, 9.44-28.16 months; *P* = .01) (Figure). In multivariable analysis, *ERBB2*-low expression remained associated with an inferior PFS (hazard ratio [HR], 1.96; 95% CI, 1.03-3.75; *P* = .04) after adjusting for line of treatment (HR for first line vs beyond first line, 0.30; 95% CI, 0.18-0.53; *P* < .001), progesterone receptor status (HR for positive vs negative, 1.48; 95% CI, 0.62-3.50; *P* = .38), and disease extent (HR for bone-only disease vs extraosseous disease, 0.50; 95% CI, 0.26-0.97; *P* = .04).

**Figure.**
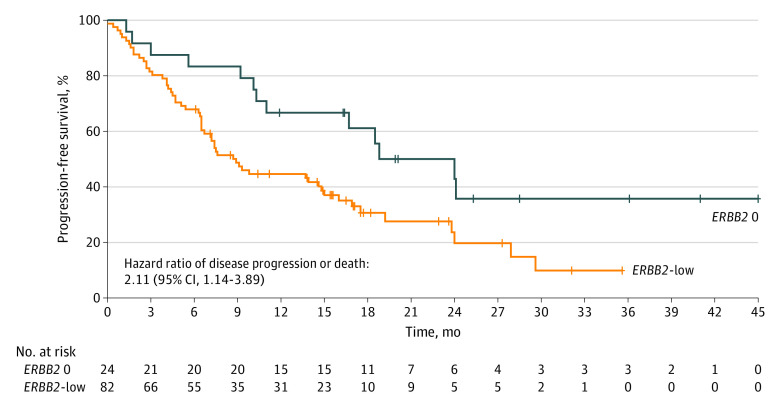
Kaplan-Meier Survival Analyses With Log-Rank Test for Progression-Free Survival *ERBB2* 0 indicates an *ERBB2* immunohistochemistry score of 0; *ERBB2*-low, an *ERBB2* immunohistochemistry score of 1+ or 2+ with negative in situ hybridization.

## Discussion

Among patients with HR+/*ERBB2*− MBC treated with CDK4/6 inhibitors, we observed that *ERBB2*-low expression was associated with an inferior PFS. This may serve as a potential marker candidate associated with CDK4/6 inhibitor efficacy. An intrinsic subtypes genomic analysis of the MONALEESA studies^3^ found that, overall, the *ERBB2*-enriched (formerly *HER2*-enriched) subtype was associated with 2.3-fold increased risk of disease progression compared with the luminal A subtype. Our results provide phenotypical evidence suggesting the inferior efficacy of CDK4/6 inhibitors in the *ERBB2*-low expression subgroup. Some limitations of this study include its retrospective nature with limited sample size, the lack of patients receiving abemaciclib, and the lack of data on *PIK3CA* mutation status. Given that novel anti-*ERBB2* antibody-drug conjugates, such as trastuzumab deruxtecan, demonstrated clinical efficacy in *ERBB2*-low–expressing MBC,^4^ coupled with emerging evidence supporting the combination use of CDK4/6 inhibitors with anti-*ERBB2* agents,^5^ this subgroup may be of high clinical relevance and warrant prospective evaluations in future trials.
